# Effects of evening light conditions on salivary melatonin of Japanese junior high school students

**DOI:** 10.1186/1740-3391-2-4

**Published:** 2004-08-11

**Authors:** Tetsuo Harada

**Affiliations:** 1 Laboratory of Environmental Physiology, Faculty of Education, Kochi University, Kochi 780-8520, Japan

## Abstract

**Background:**

In a previous study, when adult subjects were exposed to a level of 400 lux light for more than 30 min or a level of 300 lux light for more than 2 hours, salivary melatonin concentration during the night dropped lower than when the subjects were exposed to dim illumination. It was suggested that such light exposure in adolescents or children during the first half of subjective night in normal life might decrease the melatonin level and prevent the falling into sleep. However, there has been no actual study on the effects of light exposure in adolescents.

**Methods:**

Effects of exposure to the bright light (2000 lux) from fluorescent light bulbs during a period of three hours from 19:30 to 22:30 in one evening were examined on evening salivary melatonin concentrations from 19:45 to 23:40. The control group was exposed to dim light (60 lux) during these three hours. Both the dim light control group [DLCG] and the bright light experimental group [BLEG] consisted of two female and three male adolescent participants aged 14–15 y.

**Results:**

The salivary melatonin level increased rapidly from 3.00 pg/ml at 21:45 to 9.18 pg/ml at 23:40 in DLCG, whereas it remained at less than 1.3 pg/ml for the three hours in BLEG. Melatonin concentration by BLEG at 22:30 of the experimental day was lower than that at the same time on the day before the experimental day, whereas it was significantly higher in the experimental day than on the day before the experimental day in DLCG.

**Conclusions:**

Bright lights of 2000 lux and even moderate lights of 200–300 lux from fluorescent light bulbs can inhibit nocturnal melatonin concentration in adolescents. Ancient Japanese lighting from a traditional Japanese hearth, oil lamp or candle (20–30 lux) could be healthier for children and adolescents because rapid and clear increase in melatonin concentration in blood seems to occur at night under such dim light, thus facilitating a smooth falling into night sleep.

## Background

Night sleep duration of Japanese children aged 10–18 y has become shorter by one hour during the last 30 years in Japan [1]. The so called "24-hour society", which is currently in progress in Japan, seems to change environmental conditions surrounding children. For example, mobile phones are used by more than 90 % of university students and more than 30% of junior high school students living in the urban area of Kochi city (33°N) [2]. Students can communicate with their colleagues even in the middle of the night with mobile phones. Frequent or long-time (more than 30 min) usage of the mobile phone makes university and junior high school students more evening-typed [2].

"Convenience stores" are open for 24 hours and provide several kinds of food and other goods for general civilian life. Convenience stores are now common all over Japan even in suburban areas. Illumination inside the convenience stores is very bright (2000 lux or more at the level of the eyes). Bright lighting in retail stores seems to be a merchandising technique which has been in use worldwide for at least 60 years. Unconscious use of bright light in the evening or at night inside the convenience store may promote a circadian phase delay in students exposed to the bright light during the first half of subjective night. This hypothesis is based on a "light-pulse" experiment in the laboratory [3] as follows. Adult subjects were exposed to light pulse of 4000–6000 lux for 30 min at one of several phase points of their circadian rhythm, and the direction (advance or delay) and the extent of phase shift caused by the pulse were measured at each phase point. The light pulse delayed the phase of sleep-wake cycle by the subjects when they were exposed to the light pulse in the first half (about 19:00–24:00) of subjective night. However, it advanced the phase effectively when the subjects were exposed to it within three or four hours after the minimum point of inner body temperature (about 5:00–9:00). An epidemiological study about the effect of the convenience store usage was conducted on sleep habits and diurnal rhythm by about 500 students attending junior high school aged 12–15 y in Kochi Japan [4]. This latter paper reported the following three points: (1) Students going to convenience stores after sunset were more evening-typed and showed shorter night sleep of 7.0 hours on average than those going to convenience stores during the daytime, who showed night sleep of 7.3 hours on average, (2) Students who went to convenience stores every day slept only 6.4 hours on average and the sleep hours were significantly shorter than the 7.5 hours shown by students who went to convenience stores only 0–1 time per week, (3) Students who stayed more than 30 min in convenience stores took shorter night sleep of 6.6 hours on average than those who stayed there less than 15 min. (7.3 sleep hours). Younger children attending kinder garden and students attending elementary school were more sensitive to "light conditions" in normal life than university students, according to an epidemiological study [5]. However, no experimental field studies on the effects of "light conditions" during normal life have been conducted on sleep-wake cycles of healthy children younger than 15 y.

Melatonin, which is synthesized in the pineal organ and secreted to the blood, is well known as a key substance which may be effective in promoting the falling into night sleep by humans [6]. Blood melatonin concentration by adult human subjects is extremely low during daytime and increases rapidly at 22:00–23:00 up to as much as ten or twenty times daytime values. The high level is maintained till the early morning and then decreases again rapidly to the extremely low concentration typical of the daytime [7,8]. The increase in melatonin concentration might occur in late evening and trigger the falling into sleep also for healthy children, although there have been no studies on melatonin concentration in salivary or blood under their normal life. In adult subjects, a single administration of 5 mg of melatonin at 13:00 was reported to induce higher subjective sleepiness during the following 2 hours and also higher EEG power density in the range of relatively low frequency of 5.25–9.0 Hz rather than that of placebo [9]. When adult subjects were exposed to 400 lux lights for more than 30 min or exposed to 300 lux lights for more than 2 hours, melatonin level during the night became lower than that when they stayed under dimmer lights [10,11]. In the case of adolescents and children, the exposure to lights of 300 lux or more during the first half of subjective night in the normal life might decrease their melatonin level and prevent the falling into sleep.

Currently, more than 80% of junior high school students of the third grade aged 14–15 y in Kochi go to private school in the evening. If they take a short stop at the convenience store to get some fast food and enjoy talking with their colleagues in front of the store before or after going to the private school in the evening, they suffer the double exposure to bright lights at the school and at the convenience store. Such bright lights are from fluorescent light bulbs and include blue or blue-green lights with 470–500 nm wave lengths which were reported to be powerful to suppress melatonin concentration [12]. Based on the epidemiological studies made in 2001–2003 on junior high school students in Kochi Prefecture (33°N), 38.8% of the students who frequented convenience stores went there after sunset, and 30.2% and 6.5% of junior high school students who used convenience stores went there and stayed there for 15–30 min and longer than 30 min, respectively. Moreover, this epidemiological study showed that 62.4% and 18% of the students who went to the evening private school studied there for 2 and 3 hours until 9 or 10 o'clock in the evening, respectively. In total, junior high school students were estimated to be exposed to bright lights of more than 2000 lux inside private school and/or convenience store for 2–3.5 hours on average in the evening. Such exposures are expected to suppress the increase in blood melatonin level as a direct effect and also delay the phase of their circadian systems driving melatonin secretion rhythm and sleep-wake cycle.

In this study, two light conditions were investigated. One was bright and high color-temperature light of more than 2000 lux, which is used in the evening and at night inside the convenience stores, at preliminary and private school for entrance examination, and at rental video shops in Japan. The other condition was a dark and low color-temperature light with less than 60 lux which is usual in the evening for traditional Japanese settings (a fireplace, candle, or a naked light bulb).

## Methods

### Participants

Experimental participants were ten Japanese junior high school adolescent students (4 females and 6 males) aged 14–15 y who were attending Motoyama junior high school located in the mountain area of Reihoku district (33.5°N) in Kochi Prefecture. They had enjoyed New Year holidays for 7 days before the experiment. They were instructed to keep usual diurnal rhythm (for example bed time and wake-up time) during the holidays. Before the experiment, participants were divided into the two groups of "bright light experimental group (BLEG)" and "dim light control group (DLCG)". Participants in BLEG were selected to show similar circadian typology to those in DLCG based on the scores in the morningness-eveningness (M-E) questionnaire of Torsvall and Åkerstedt [13] (mean ± SD: 15.00 ± 4.30 by BLEG and 14.80 ± 4.09 by DLCG). Bed time, wake-up time and sleep hours shown by BLEG for the four days just before the experiment were 23.0 ± 4.2 hours, 8.4 ± 1.9 hours and 9.1 ± 1.4 hours, respectively; corresponding values for DLCG were 23.8 ± 1.3 hours, 8.9 ± 1.3 hours, and 9.5 ± 1.5 hours. Each group consisted of two females and three males. All the ten participants sampled their own saliva using "Salivette" collecting tubes (SARSTEDT Aktiengesellschaft & Co., Numbrecht, Germany) at 22:30–23:00 under the 200–300 lux light from fluorescent light bulbs in their home on the day before the experimental day.

Japanese civilians seem to enjoy evening time during the first half of subjective night (after sunset till bedtime) under fluorescent light bulbs based on our unpublished questionnaire study on 950 families having small children aged 0–6 yrs in Kochi. More than 85% of the 950 families enjoyed evening life under fluorescent light bulbs. We measured the illumination at the level of 1 m above floor just under a usual type of round-shaped fluorescent light bulb in a typical one-room apartment for students and it was 340 lux.

### Procedure

On the experimental day of the 5^th ^January 2003, all the ten participants got together in front of Motoyama junior high school at 8:00 in the morning. A wagon car took them to the experimental place which was a Japanese style hotel located at a mountain area, Yusuhara town in Kochi Prefecture, 126 km west from Motoyama town. During the driving, illumination inside the car was 350–500 lux. The car arrived at the hotel around noon. It was snowing through the day. Behavior of all the participants was controlled during the stay in the hotel till the next morning of the experimental day. All the participants played outside exposed to the sun light with 6000–7500 lux at the eye level during 12:30–13:30 and 14:00–14:50. They were allowed to have a rest in a living room in which the floor was filled with 12 *tatami *mats and the illumination at the eye level was 250 lux from fluorescent light bulbs during the rest of the time till 16:30. Participants took bath one by one between 16:30–18:00 and had supper all together between 18:15 and 19:20 in the living room. At 19:25, the participants of BLEG moved to a Japanese style room with 8 *tatami *mats where they were exposed to the light with 2000 lux at the eye level from fluorescent light bulbs, whereas DLCG group members moved to another Japanese style room with 8 *tatami *mats where they were exposed to the light of 60 lux and relatively low color-temperature from a electronic light bulb. All the participants included in both groups were home-working or making a small wooden folk craft object that is typical in the Yusuhara district, under each light condition till 22:30. Room temperature was controlled at 15 ± 2°C with an oil heater in both groups. Then they came back to the former living room (12 *tatami *mats) and stayed there under the light of 250 lux till 23:40. Then female and male participants moved to separate rooms and went to bed just before 24:00. Salivary samples were collected in collection tubes at 21:45, 22:30, and 23:40, and these salivary samplings were preserved in a refrigerator at less than -20°C. Melatonin concentration in the samples was analyzed by a professional analyzing company (MSL Co. Ltd.) which was a specialist for several chemical and microbiological analyses. All the participants from both groups were called out to get up at 7:00 in the next morning. All the participants got up between 7:00–7:15 responding to the calling out. After taking breakfast, they left the experimental place at 9:00 back for Motoyama junior high school. Throughout the study, light exposure was measured on the eye level with a digital illumination meter.

Detailed explanation of the objectives and methods of the experiment was provided before the experimental performance to the participants and their parents. The research project received full and complete agreement from all of them.

## Results and Discussion

**Figure 1:**
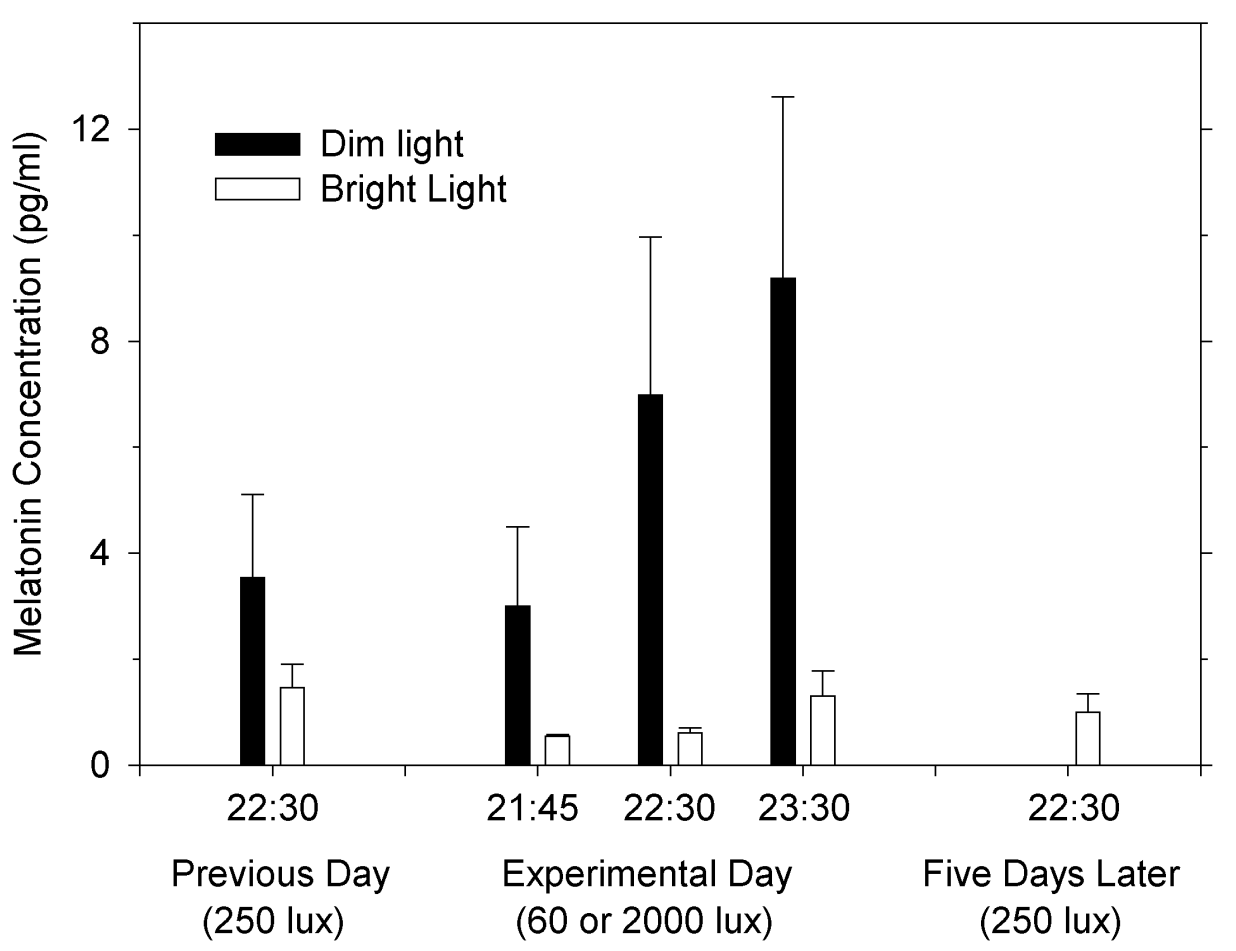
**Effects of light condition on salivary melatonin concentration. **Values shown are means (n = 5 per group) and SEM.

The results are shown in Fig. 1. Salivary melatonin concentration rose from 3.00 ± 3.34 (mean ± SD) pg/ml at 21:45 to 9.18 ± 7.66 pg/ml at 23:30 of the experimental day in the DLCG (t-test between values at 21:45 and 23:30: t = 3.60, df = 4, p < 0.05), whereas it remained at less than 1.3 pg/ml till 23:30 in BLEG (t = 2.07, df = 4, p < 0.2). There was no significant difference in the melatonin concentration between BLEG and DLCG in the day before the experimental day (Wilcoxon test: z = -1.163, p = 0.31). At 22:30 of the experimental day, melatonin concentration by BLEG tended to be lower than that on the day before the experimental day (Wilcoxon test: z = -1.604, p = 0.109), while the concentration became significantly higher in DLCG (z = -2.023, p = 0.043). On the day before the experimental day, all the participants were under Japanese standardized light condition with 200–400 lux from a fluorescent light bulb with relatively high color-temperature. On the experimental day, the bright light of 2000 lux in BLEG suppressed the expected night increase of melatonin concentration, whereas the relatively low color- temperature light with 60 lux did not.

In Japan, bright and high color-temperature light of more than 2000 lux is available in the evening or night inside convenience stores which are open for 24 hours and private schools for the preparation to go through the entrance examination to upper schools. Also in usual life, such exposures to bright lights in the evening private school and convenience store can suppress the night increase in blood melatonin level as a direct effect and possibly delay the circadian system that drives the melatonin secretion rhythm and sleep-wake cycle.

The results of this study suggest that ancient Japanese lighting in the evening and at night, which could be supplied by a traditional Japanese hearth fire or a oil lamp or candle (20–30 lux), might be healthy for adolescents and children, because the ancient lights could allow rapid and clear increase in melatonin level leading to a smooth falling into night sleep [14].

## Conclusions

Bright lights of 2000 lux and even moderate lights of 200–300 lux can inhibit, as a direct effect, nocturnal melatonin concentration in children. Ancient Japanese light conditions which could be supplied by a traditional Japanese hearth fire or a small oil lamp or candle might be healthy for children, because the ancient lights could allow rapid and clear increase in melatonin level in the evening, leading to a smooth falling into night sleep.

## Competing interests

None declared.
